# Supplementary material for: The Effect of Fractional Composition on the Graphite Matrices’ Porosity
Source: Materials (Basel). 2024 Oct 24;17(21):5171. doi: 10.3390/ma17215171 (PMC11547188; doi:10.3390/ma17215171)
Supplement: Supplementary file 1 [file materials-17-05171-s001.zip › materials-3231124-supplementary.pdf]

Table S1. Geometric parameters of graphite powder samples without the addition of phenolic resin at different pressures

Pressure, MPa	Height, mm	Diameter, mm
5	12.54	26.32
10	14.91	26.00
15	14.64	26.00
20	13.90	26.43
25	13.40	26.79
30	13.12	26.18
35	12.23	26.41
40	12.69	25.85
45	12.00	26.16
50	11.71	25.53
55	11.73	25.74
60	11.00	26.44

Table S2. Geometric parameters of phenolic resin samples different pressures

Pressure, MPa	Height, mm	Diameter, mm
5	4.89	12.41
10	4.96	12.24
20	5.32	11.77
35	5.32	11.01

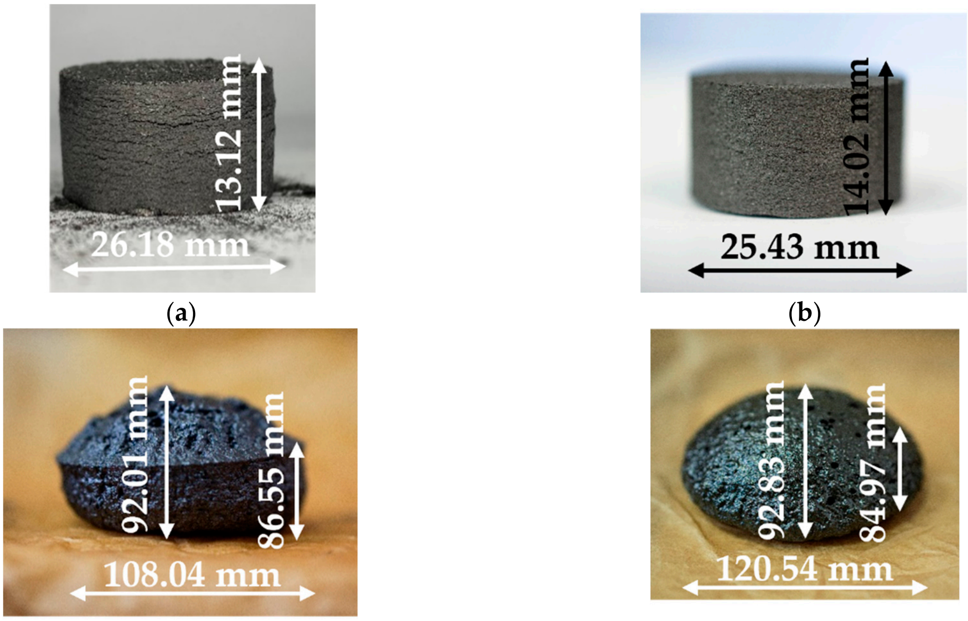

(a) (b) (c) (d)

Figure S1. Binder content impact on the geometric parameters of graphite preforms after thermal treatment: (**a**) 0% phenolic resin; (**b**) 40% phenolic resin; (**c**) 50% phenolic resin; (**d**) 60% phenolic resin.

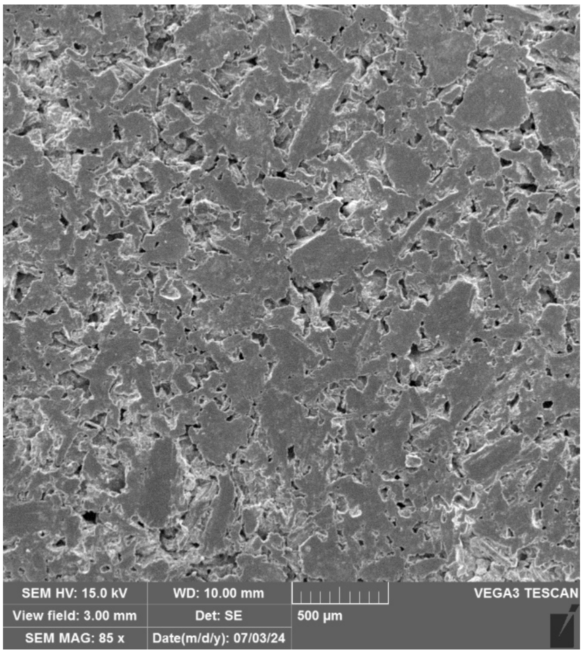

(**a**)

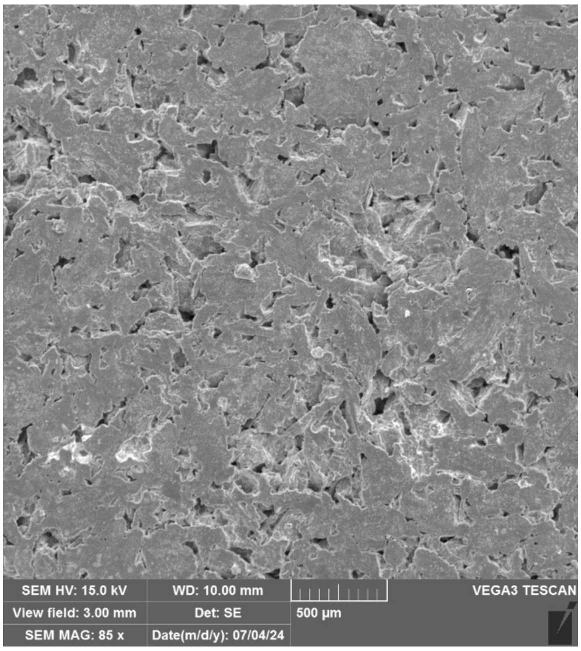

(**b**)

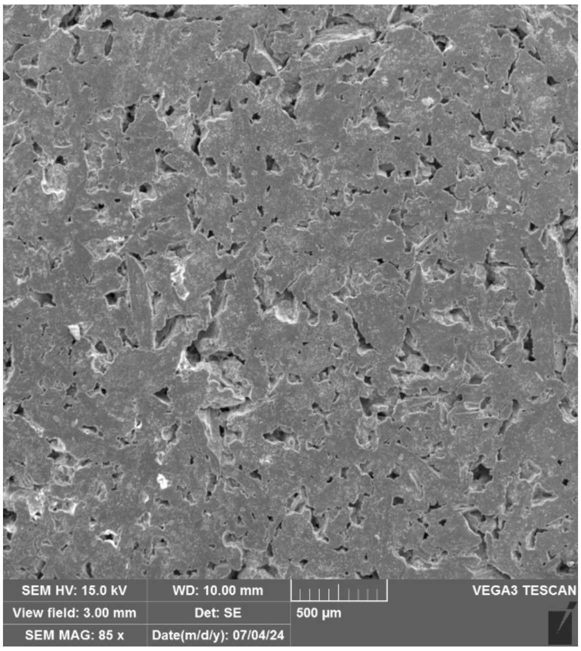

(**c**)

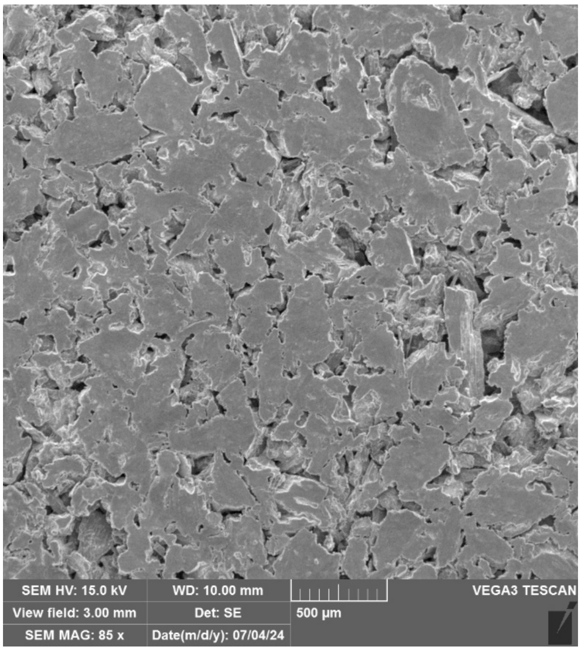

(**d**)

Figure S2. SEM images of graphite matrices with usual phenol resin – 20% (mass) content: (a) with granulometric distribution 50 %- 63-100 μm, 25% - 250-315 μm, 25% - 100-250 μm; (b) with granulometric distribution 50%- 100-250 μm, 25% - 63-100 μm, 25% - 259-315 μm; (c) with granulometric distribution 50% - 250 – 315 μm, 25% - 63-100 μm, 25 %- 100-250 μm; (d) with granulometric distribution 33% - 100-250 μm, 33% - 63-100 μm, 33% - 259-315 μm.

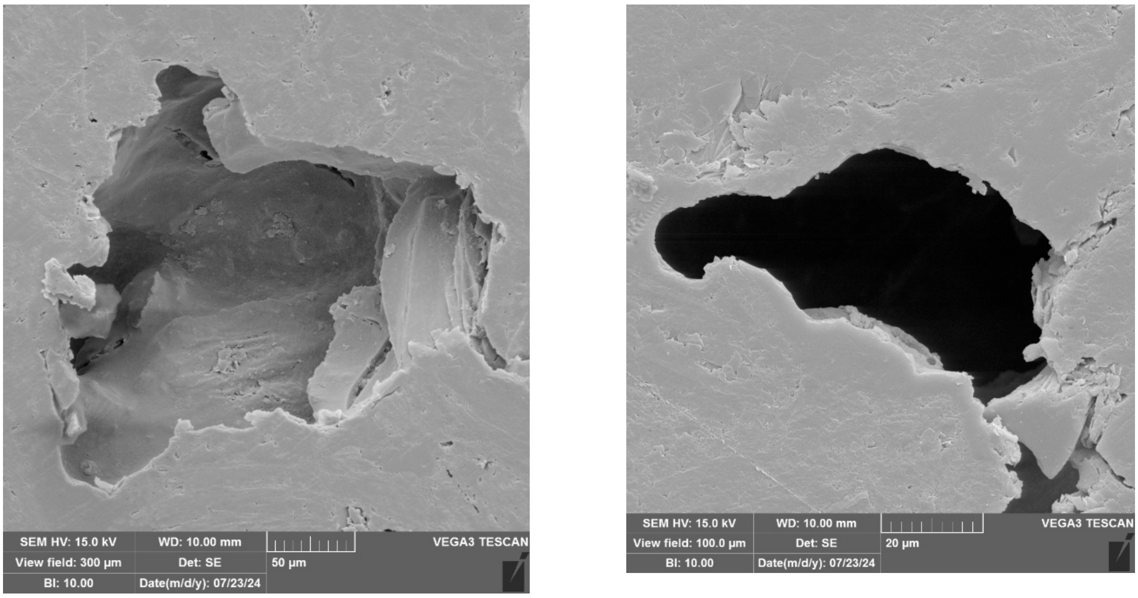

(**a**) (**b**)

Figure S3. SEM images of pores in a graphite matrix after carbonization: (**a**) the pore is filled with amorphous carbon; (**b**) graphite pore.

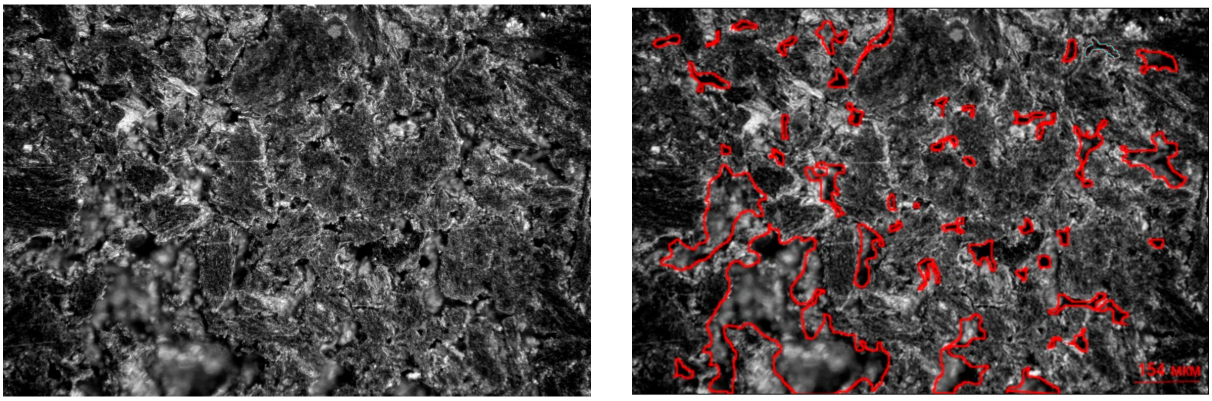

(**a**) (**b**)

Figure S4. Optical image of a graphite matrix surface with 50% 63-100 μm at magnification ×10: (**a**) general view; (**b**) with highlighted pores. The average areal porosity is 10.93%.

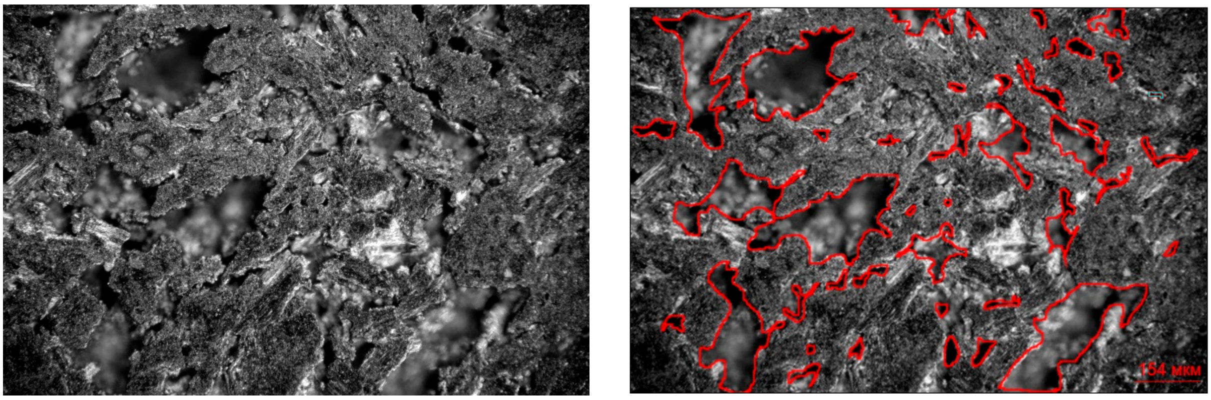

(**a**) (**b**)

Figure S5. Optical image of a graphite matrix surface with 50% 100-250 μm at magnification ×10: (**a**) general view; (**b**) with highlighted pores. The average areal porosity is 19.77%.

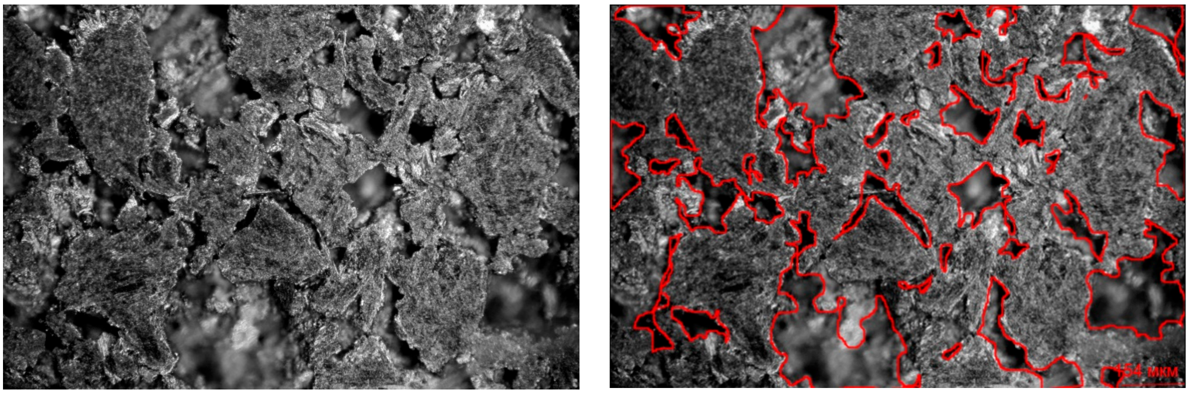

(**a**) (**b**)

Figure S6. Optical image of a graphite matrix surface with 50% 250-315 μm at magnification ×10: (**a**) general view; (**b**) with highlighted pores. The average areal porosity is 18.19%.

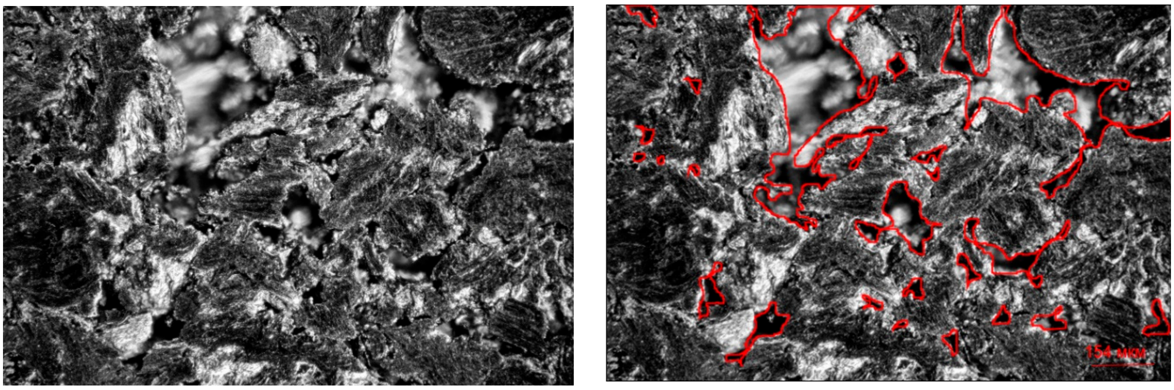

(**a**) (**b**)

Figure S7. Optical image of a graphite matrix surface with 33% of all fractions at magnification ×10: (**a**) general view; (**b**) with highlighted pores. The average areal porosity is 12.54%.

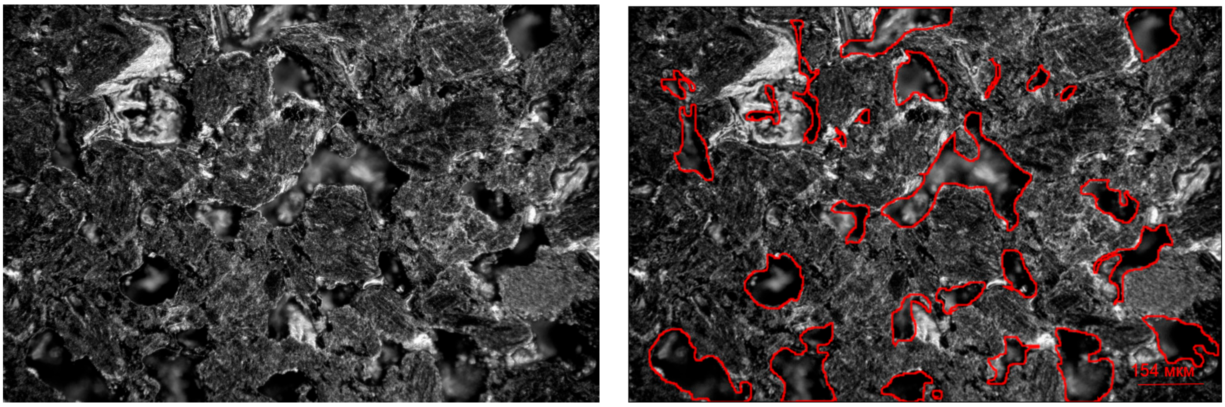

(**a**) (**b**)

Figure S8. Optical image of the surface of a graphite matrix with an excessive content of phenol resin (40%) and graphite fraction 315 μm at magnification ×10: (**a**) general view; (**b**) with highlighted pores. The average areal porosity is 12.09%.
